# Zinc oxide nanorods functionalized paper for protein preconcentration in biodiagnostics

**DOI:** 10.1038/srep43905

**Published:** 2017-03-02

**Authors:** Sadhana Tiwari, Madhuri Vinchurkar, V. Ramgopal Rao, Gil Garnier

**Affiliations:** 1 Department of Electrical Engineering, Indian Institute of Technology Bombay, Mumbai, 400076, India; 2 BioPRIA, Chemical Engineering department, Monash University, Clayton VIC 3800, Australia

## Abstract

Distinguishing a specific biomarker from a biofluid sample containing a large variety of proteins often requires the selective preconcentration of that particular biomarker to a detectable level for analysis. Low-cost, paper-based device is an emerging opportunity in diagnostics. In the present study, we report a novel Zinc oxide nanorods functionalized paper platform for the preconcentration of Myoglobin, a cardiac biomarker. Zinc oxide nanorods were grown on a Whatman filter paper no. 1 via the standard hydrothermal route. The growth of Zinc oxide nanorods on paper was confirmed by a combination of techniques consisting of X-ray diffraction (XRD), X-ray photoelectron spectroscopy (XPS,) scanning electron microscopy (SEM), and energy dispersive spectroscopy (EDX) analysis. The Zinc oxide nanorods modified Whatman filter paper (ZnO-NRs/WFP) was further tested for use as a protein preconcentrator. Paper-based ELISA was performed for determination of pre-concentration of cardiac marker protein Myoglobin using the new ZnO-NRs/WFP platform. The ZnO-NRs/WFP could efficiently capture the biomarker even from a very dilute solution (Myoglobin < 50 nM). Our ELISA results show a threefold enhancement in protein capture with ZnO-NRs/WFP compared to unmodified Whatman filter paper, allowing accurate protein analysis and showing the diagnostic concept.

Many of the point-of-care hand-held devices are based on the detection of very low concentrations of some specific protein biomarker in a blood or biofluid sample. There is often a need to preconcentrate the analyte prior to measurement on the sensing area of the test to enhance the detection sensitivity for these miniaturized devices. Many of the current systems rely on high voltage driven electrokinetic or isotachophoretic protein preconcentrators and are typically membrane based devices requiring expensive and complex microfabrication techniques to manufacture^1^,^2^,^3^,^4^. Paper-based devices provide an increasingly popular alternative in diagnostics and environmental monitoring as they are inexpensive, easy to fabricate and to modify, and once used, easy to dispose as they easily burn and are biodegradable. Paper tests rely on the inherent capillary force created by the network of paper hydrophilic pores/fibers in which aqueous fluids naturally wick; no external driving force or systems are required for fluid transport. Paper also provides a good support for growing nanostructures by providing a template for orientation and nucleation sites. This has been demonstrated in previous studies including our attempt to grow ZnO nanorods on paper by various protocols^5^,^6^,^7^,^8^. Paper has been widely used as substrate for many μPAD devices and blood plasma separation^9^,^10^, there are very few studies reporting the use of paper devices for protein preconcentration^11^,^12^,^13^.

Recently, paper has been used creatively in many sensors^14^,^15^, including blood typing test^16^, point of care diagnostic^17^,^18^ and environmental monitoring sensor^19^. Biocompatibility and biodegradability makes it suitable for many low-cost strip based assays^20^,^21^. While the first generation of paper-based analyses was mostly based on colorimetric detection^22^,^23^, more specific selective or sensitive detection techniques including electrochemical^24^,^25^,^26^,^27^, piezoelectric and piezoresistive detection^28^,^29^,^30^ have recently been reported relying on paper modification with nanomaterials. To preconcentrate an analyte using paper as substrate represents a limiting step for many applications. A few research groups have investigated paper as a platform to capture, concentrate and quantify low concentrations of a desired analyte from large sample volumes^12^,^13^,^31^. However, none has investigated the promising potential nanotechnology as a mean to increase surface area and selectivity. The efficient capture of a low abundance target protein requires a preconcentrator combining either a high specificity for the targeted protein or a very large surface area; this can be achieved by growing 1-D nanostructures on paper and functionalizing those for high adsorption specificity for the marker of interest. In a second step, the adsorbed protein is desorbed from the nanostructured paper into the solution by providing a step change in its pH, ionic strength or even temperature. This approach provides a promising strategy to capture and detect very low concentration marker protein in biosamples. Solution-phase grown 1-D ZnO nanostructures like nanorods, nanobelts and nanotubes are attractive for optoelectric devices and sensors due to their functional properties^32^,^33^; various ZnO nanostructures are already used in biomedical and other fields^34^,^35^. ZnO-nanostructures of different morphology could also serve as sensor to elucidate anticancerous and virostatic mechanisms^36^,^37^,^38^,^39^,^40^,^41^,^42^,^43^. One such protein preconcentrator based on silicon nanowires grown on a silicon dioxide wafer showed to efficiently preconcentrate and sense specific protein from whole blood sample^44^. However, this preconcentrator required both expensive materials and microfabrication.

Here, we report for the first time a paper modified with metal oxide nanorods as an efficient protein preconcentrator platform to produce a low-cost and biodegradable sensor. Zinc oxide nanorods were grown on a Whatman filter paper no.1 via the standard hydrothermal route^5^. Paper substrate favors the growth of oriented micro or nanostructures due to the inherently oriented and organized cellulose fiber network^6^. Nanostructures based on Zinc oxide (ZnO) have excellent biocompatibility are non-toxic, chemically stabile and have a high isoelectric point i.e. 9.5 45; they are ideal for the immobilization of biomolecules^46^,^47^,^48^.

In this study, we report a novel application of the ZnO nanostructure-paper composite as myoglobin protein preconcentrator. Our results show ZnO nanorod modified paper based platform to efficiently capture the target protein biomarker from dilute and large volume sample. The successful release of the captured protein without loss in activity was demonstrated. Further validation of preconcentration was performed using paper-based ELISA (P-ELISA) for cardiac myoglobin on ZnO-NRs/WFP. While a few research groups have investigated P-ELISA as analytical technique for protein concentration determination^49^,^50^,^51^, there is no report of ZnO-nanostructure modified paper engineered as preconcentrator for biosensor application.

## Results

### Morphological characterization of ZnO-NRs grown on Paper

**Figure 1:**
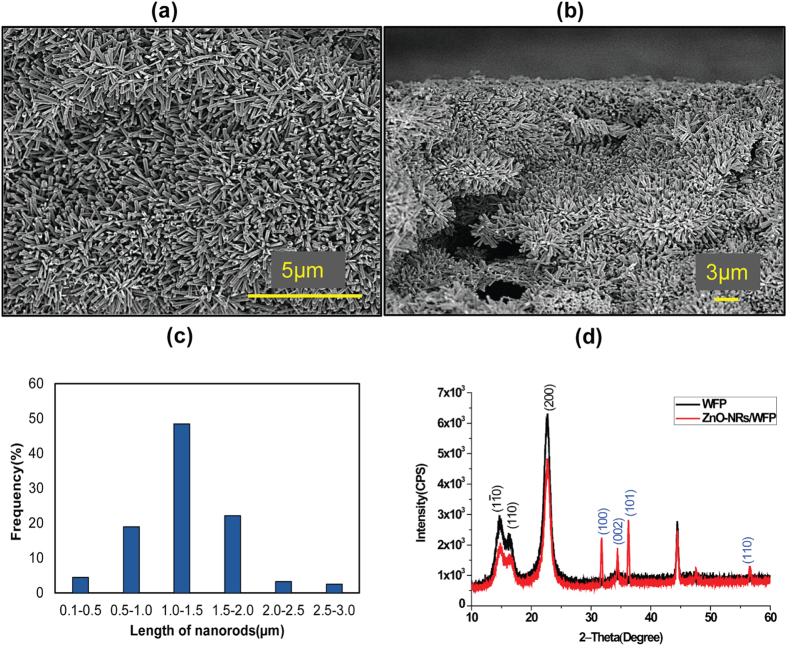
SEM images of ZnO-NRs/WFP. (**a**) Top view of the modified paper showing surface coverage by the nanorods, (**b**) cross section of the ZnO-NRs/WFP, (**c**) Histogram showing average length of nanorods grown on paper, (**d**) XRD spectra of WFP; without ZnO-NRs (black) and with ZnO-NRs (red).

Hydrothermally grown Zinc Oxide nanorods (ZnO-NRs) on Whatman filter paper were characterized by a series of complementary techniques. The surface morphology and surface coverage of the sample were observed by scanning electron microscopy (SEM). Figure shows scanning electron micrographs of zinc oxide nanorods functionalized paper representing the growth pattern of the nanorods on paper fibers (Fig. 1a) and the cross-sectional SEM micrograph indicating that the nanorods grew all through the paper with very good surface coverage (Fig. 1b). The average length of the nanorods was 1 μm with an average diameter of 200 nm. Figure 1c shows the length distribution of nanorods (calculated from 400 nanorods).

To characterize the crystallinity of the modified paper, X-ray diffraction technique was used and the recorded XRD diffractogram of a ZnO-NRs/WFP, presented in Fig. 1d, exhibits the diffraction pattern characteristic of hexagonal ZnO with peaks corresponding to the (100), (002), (101) and (110) crystallographic planes, positioned at 2θ = 31.7°, 34.4°, 36.2° and 56.6°. This is consistent with results previously reported on ZnO films and ZnO nanostructures^5^,^52^. The (002) reflection peak indicates film with crystal growth with a preferred orientation along the c-axis. Diffraction peaks at 2θ = 14.7°, 16.8° and 22.7° correspond to the planes of crystallinity of cellulose forming the fibers of the Whatman filter paper.

### Elemental analysis of ZnO-NR functionalized Paper

**Figure 2:**
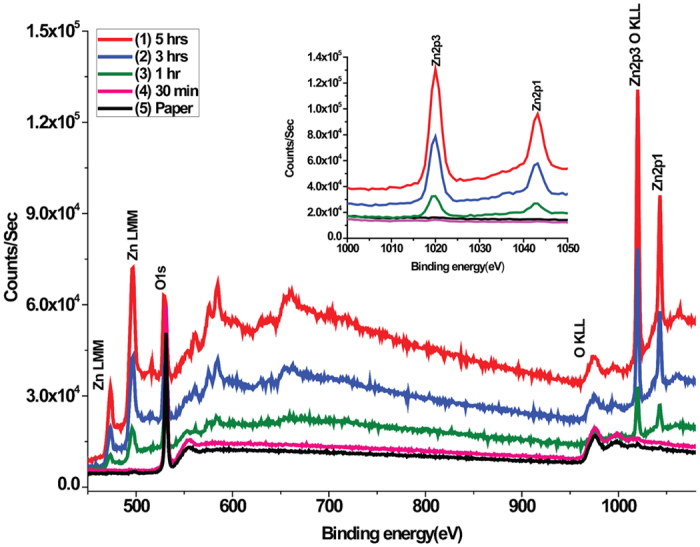
XPS survey spectra showing the growth dynamics of ZnO-nanorods on paper at different time and highlight of the peaks specific to ZnO formation (inset).

Figure 2 shows the full XPS spectra of WFP functionalized with zinc oxide nanorods at different growth time. The spectral peak intensities reveal that the formation of ZnO linearly increases with growth time. The graph in inset shows the specific peak enhancement of Zinc as a function of growth time. The penetration depth of the X-ray used was 7 nm. XPS spectra also show the effect of process time on the atomic concentration of Zinc. The characteristic peak of ZnO formation (Zn2p3) is seen at 1021 eV binding energy and no metallic Zn peak was observed. After 5 hours of growth, the spectral intensity (~14 × 10^4^ c/s) is much higher than at 1 hr and 3hr. Spectra analysis shows no significance growth after 30 min (Fig. 2). Elemental heterogeneity of the modified paper was quantified using EDX. The EDX spectra of ZnO-NRs/WFP provide a detailed picture of elemental composition and their contribution to the surface. (Supporting information, S1).

### Antibody immobilization

**Figure 3:**
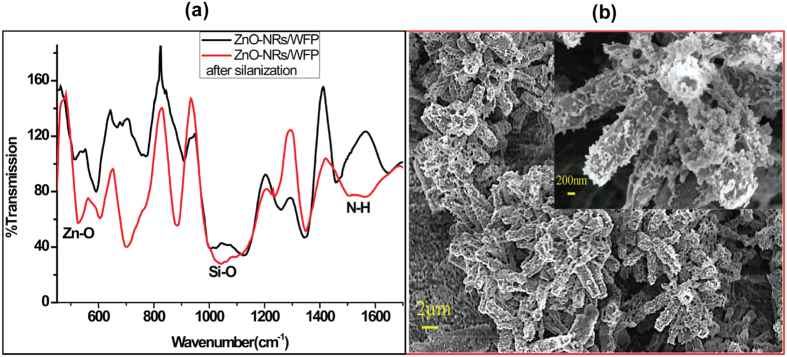
FTIR-spectra (**a**) and SEM image (**b**) of ZnO-NRs/WFP after APTES modification. The inset shows the magnified micrograph of ZnO-nanorods after antibodies immobilization.

The ZnO-NRs/WFP modified paper was biofunctionalized with antibodies. The antibodies were covalently immobilized using silanization chemistry with 3-APTES, which provides sites for amide(–CONH_2_) bond formation. Silanization of ZnO-NRs/WFP with 3-APTES was confirmed by FTIR (Fig. 3a). The peak at about 3410 cm^−1^ corresponds to the stretching vibration of N–H and O–H. The peaks at about 1578 and 1517 cm^−1^ are attributed to the bending vibration of N–H which is absent for paper without silanization. The peak at 1016 cm^−1^ refers to the Si–O bond stretching vibration, while at 493 cm^−1^ there is clear peak in modified paper which belongs to the Zn–O bond stretching vibration.

Silanized ZnO-NRs/WFP paper was then biofunctionalized with the antibodies. A typical SEM image of the biofunctionalized ZnO-NRs/WFP (Fig. 3b) shows the immobilized antibodies on nanorods.

### Confocal microscopy of ZnO-NRs functionalized Paper

**Figure 4:**
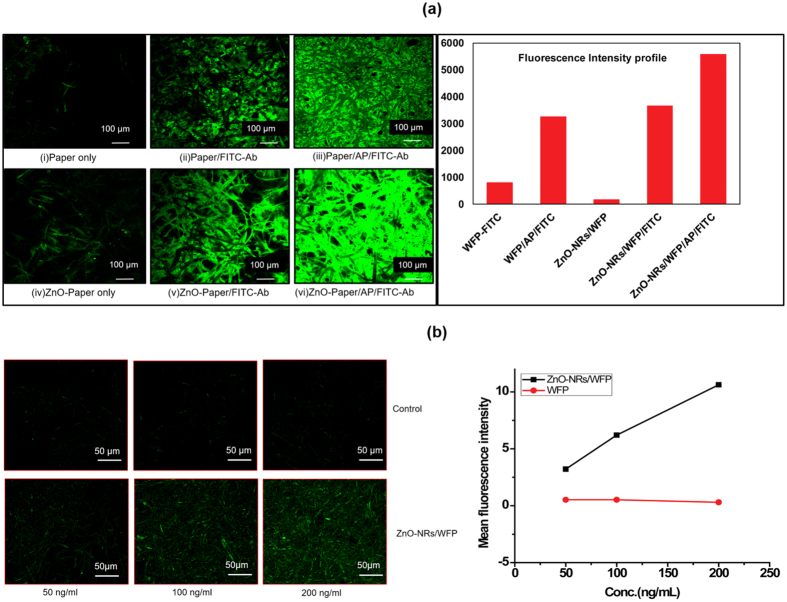
(**a**) Confocal microscopy images and fluorescence intensity profile of WFP, WFP/FITC-Ab, WFP/AP/FITC-Ab as control and ZnO-NRs/WFP, ZnO-NRs-WFP/FITC-Ab, ZnO-NRs-WFP/AP/FITC-Ab; (**b**) Images with low protein concentration on ZnO-NRs/WFP and WFP (control) and plot showing change in fluorescence intensity with concentration in both types of paper.

Confocal microscopy can measure not only surface fluorescence but also in-depth fluorescence which is very crucial to accurate quantification of the adsorbed molecules in the substrate. Figure 4 shows clear enhancement of fluorescence indicating efficient immobilization of the FITC-Ab for ZnO-NRs modified paper [Fig. 4a(vi)]. Fluorescence images for both ZnO-NRs-treated paper and filter paper strips were captured without [Fig. 4a(ii),(iv)] and with APTES (AP) coupling chemistry[Fig. 4a(iii),(vi)] to ensure that the observed fluorescence is due to binding of antibodies over the surface and not to unspecific adsorption. The extreme right of Fig. 4a is the plot of fluorescence intensity from various paper strips. Integrated intensity density of fluorescence was calculated using the same area for each image.

### Paper-based ELISA for Validation

**Figure 5:**
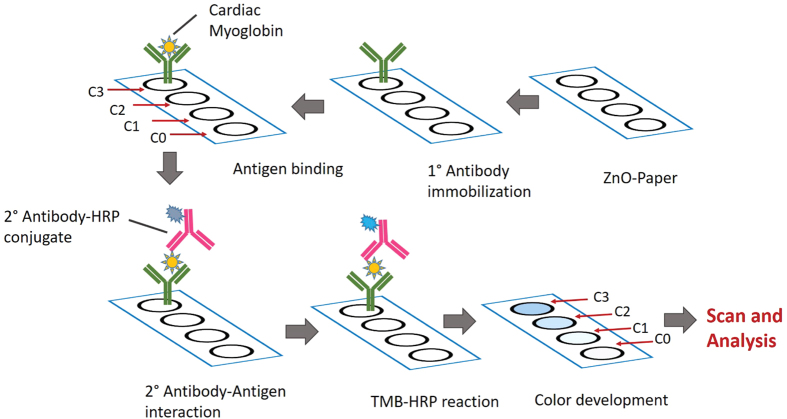
Schematic of Paper-ELISA representing the steps of the detection process. In this sandwich ELISA, identical circular regions with hydrophobic boundaries were marked on ZnO-NRs/WFP; the primary antibodies were immobilized on ZnO paper for 10 min.; four different concentrations of antigen were allowed to bind for 10 min.; HRP-conjugated secondary antibodies were allowed to bind for 1 min. followed by a quick wash with PBS and then TMB was added to react with the enzyme HRP for 30 min and produce colour. The strips were scanned and the colour intensity and spectra were analyzed using imageJ (version 1.45).

To support the results from confocal microscopy and to show the activity as well as the efficiency of the bound protein on paper, we performed paper based ELISA(P-ELISA) on ZnO-NRs/WFP. A schematic of the P-ELISA methodology performed on the strips is illustrated in Fig. 5.

**Figure 6:**
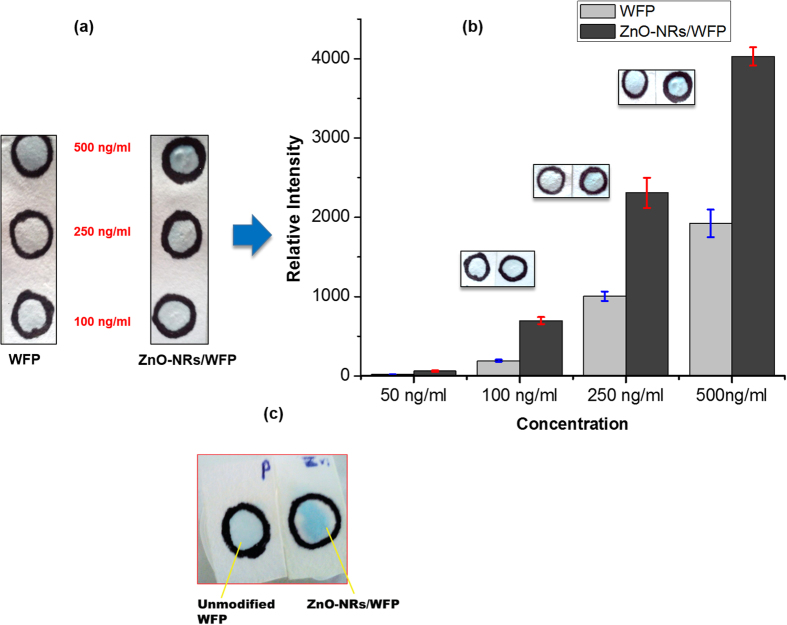
P-ELISA at different protein(myoglobin) concentration (**a**), plot showing colour intensity comparison for two types of paper using image J (**b**) and comparison with much diluted protein solution on unmodified WFP and ZnO-NRs/WFP (**c**).

Upon completion of the ELISA, the colour intensity of each test region was determined by images analysis. Concentration versus relative intensity plots obtained for three separate sets of sandwich paper-ELISA are given in Fig. 6. ZnO-NRs/WFP can capture very low concentration of cardiac myoglobin (50 nM) very efficiently compared to paper (Fig. 6a,b) due to the high available surface area contributed by ZnO-nanorods. The plot obtained from P-ELISA provides a rough quantitative estimation of bound target protein in terms of colour intensity (Fig. 6b). To evaluate if the protein capture efficiency of ZnO-NRs/WFP is retained even with large volumes, we passed 1 mL of protein solution (0.5 ng/mL) through the paper and performed the ELISA assay. Figure 6c shows the difference in colour intensities of unmodified WFP and ZnO-NRs/WFP. The enhancement of protein capture by ZnO-nanorods modified paper is demonstrated by a more intense blue colour on ZnO-NRs/WFP. The ZnO-NRs/WFP was further tested as a protein pre-concentrator platform by elution of bound protein.

### Measuring the eluted protein from ZnO-NR functionalized Paper

A semi-quantitative estimation of eluted protein was performed by measuring fluorescence spectroscopy intensity. The absorbance of eluates with FITC tagged antibodies was recorded at 488 nm and the corresponding emission at 520 nm.

**Figure 7:**
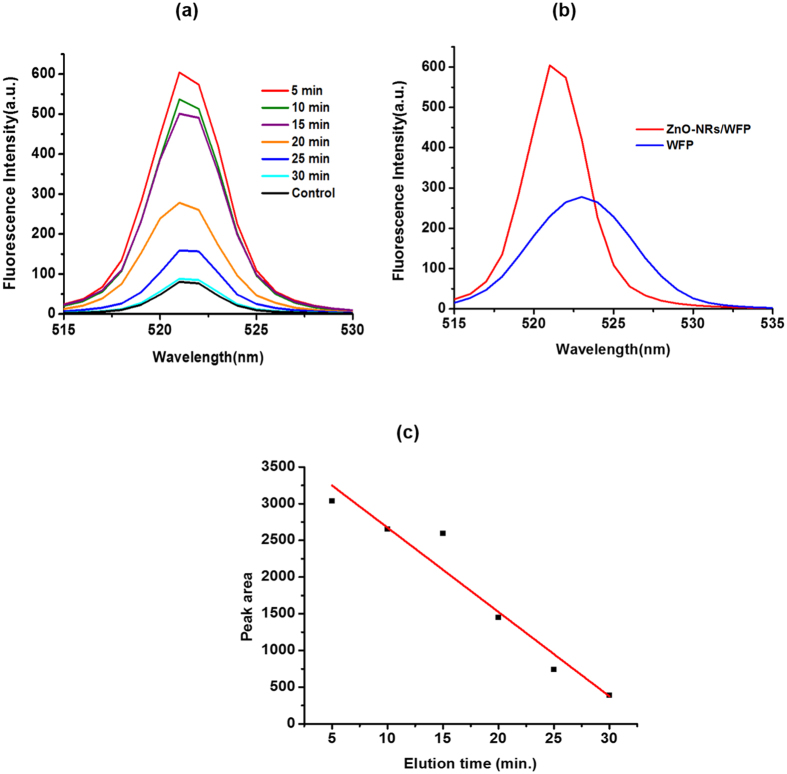
(**a**) Emission spectra for FITC-Ab after elution at different time (**b**) Comparison of fluorescence intensity of FITC-Ab with and without ZnO-nanorods on paper (**c**) Plot between area under the individual spectrum with respect to elution time.

Fluorescence spectra of the protein eluates collected from ZnO-NRs/WFP at different time intervals are presented in Fig. 7a. Most of the bound protein eluted from the substrate within 20 min. Emission spectra for the protein eluates obtained from ZnO nanorods paper and paper control are compared in Fig. 7b. The fluorescence intensity of tagged protein was higher on ZnO-NRs/WFP which corroborates the results from P-ELISA and confocal microscopy. More than two-fold increase in emission intensity of the protein eluate in the case of ZnO-NRs/WFP compared to the control (unmodified WFP) sample indicates efficient protein pre-concentration by the ZnO nanorods. The ZnO-NRs/WFP platform can now be integrated in biosensors for the detection of cardiac biomarkers.

## Discussion

ZnO-NRs modified paper has proven an efficient platform for preconcentration of proteins, which was shown with fluorescence studies and confirmed with P-ELISA. The surface coverage by ZnO-nanorods on paper was calculated from SEM micrographs to represent ~90% of the total surface area of paper. With their high aspect ratio and high surface coverage, nanorods provide a very large surface area on modified paper for binding biomolecules. In complementarity, cellulose crystallinity also plays an important role to support the growth of nanorods on paper by providing a template with hierarchical ordered structures at length scales ranging from 5 nm to the μm. Antibodies can efficiently be immobilized on the nanorods of these modified paper; however, biomolecules can also adsorb on paper because of its porous structure and by non-specific binding. To avoid this, the antibodies were covalently immobilized using APTES- chemistry which provides sites for -NH_2_ bond formation and the unreacted surface was blocked with 1% BSA. The tremendously enhanced binding of tagged antibodies with the modified paper is reflected by the confocal microscopy images obtained with fluorescence enhancement (Fig. 4a). The plot in Fig. 4 shows that ZnO-NRs/WFP without any fluorescent molecule does lightly fluoresce because of the inherent photoluminescence of ZnO at 488 nm^53^. Confocal microscopy analysis also reveals that covalent coupling of antibodies is a more effective way of biofunctionalizaton than physical adsorption; this is valid for both types of paper (ZnO nanorod modified and unmodified). Figure 4b shows that with ZnO-NRs/WFP there is some level of fluorescence even at the lowest concentration used and that fluorescence increases with the concentration of the protein solution. Whereas, with the control WFP there is no significant fluorescence observed at any of the three protein concentrations studied. The contribution of ZnO-nanorods to increase the surface area available for biomolecules immobilization was very significant thanks to their high surface coverage on paper providing enhancing area by a factor of 6.5. Image analysis combined with basic calculations show that a 100 μm^2^ (10 μm × 10 μm) section of paper, once treated, has a 90% surface coverage of hexagonal ZnO rods, consisting of 1000 rods 1 μm high and with a diagonal of 200 nm; this forest of rods provides an additional surface area of 650 μm^2^ (S2 in Supporting information). Even at low concentration of antibodies, the ZnO-nanorods modified paper shows significant fluorescence in comparison with the unmodified paper, and the fluorescence intensity increases linearly with the increase in concentration of protein. However, fluorescence does not increase linearly with surface area as the 6.5x increase of surface area provided by the ZnO nanorods only provides a 2x factor increase in fluorescence. This might be due to some quenching or fluorescence saturation effect.

P-ELISA provides quantitative estimation of bound target protein i.e. cardiac myoglobin in terms of colour intensity. An enhanced colour intensity was observed when the protein solution was flown through the ZnO-NRs/WFP as compare to the unmodified paper. For a preconcentrator, beside concentrating the target protein, the ability to release the captured protein is equally important so that it can be pass through the sensing device. Protein elution was based on breaking antigen-antibody interaction using high pH = 8.5 buffer. The pH shock created the breakage of protein-antibody interaction completely within 30 min., whereas maximum amount of bound protein was eluted in 15 min. with passing elution buffer. Post-elution, the fluorescence intensity of the tagged antibodies was higher with ZnO-NRs/WFP; this is likely due to the higher amount of captured protein with modified paper and subsequently the higher amount of eluted protein than with the untreated paper – using the in same eluted volume. The desorption rate of proteins from biospecific binding is probably higher than that from protein physically adsorbed on paper through un-specific interactions, typically less sensitive to pH. This complements the results obtained from P-ELISA and confocal microscopy. Further studies are needed to investigate the performance of the ZnO-NRs modified paper platform with real sample like; blood, plasma and serum and its design and integration with the sensing platform.

## Conclusion

Novel ZnO nanorods functionalized paper was investigated as a preconcentrator unit for application in paper biotest and biodiagnostic devices. The efficiency of the ZnO nanorod treated paper as protein concentrator was successfully tested for myoglobin, a biomarker for heart disease. Hexagonal nanorods of high aspect ratio and very high packing density were grown on paper to provide the high surface area of binding sites required to chemisorb the antibody molecules and was used to selectively capture the target protein. In a first step, a preconcentrator is required to capture high density of the target protein for the concentrating step; in the second, the paper unit must be able to elute the captured protein so that it can be pass through the sensing device for detection. ZnO-NRs/WFP chemisorbed with an antibody proved very efficient for both steps with myoglobin full release upon change of pH (8.5). Fluorescence spectroscopic study using fluorescent tagged molecules showed the bound protein to be released from ZnO-NRs/WFP without loose in activity. P-ELISA provides quantitative estimation of bound target protein i.e. cardiac myoglobin in terms of colour intensity. Using ZnO nanorod paper as preconcentrator, myoglobin could be detected at concentration as low as 50 ng/mL. ELISA results revealed a threefold enhancement in protein capture with ZnO-NR paper compared to the control paper. This ZnO-NRs/WFP platform can easily be integrated with μ-PAD or other biosensors based on antigen-antibody interaction. Future scope of the work involves the design and integration of the preconcentrator with sensing device and experimentation with mixture of different proteins and other biomarkers in biofluid samples including blood, plasma and serum.

## Materials and Methods

All chemicals were analytical grade reagents used without further purification. Zinc acetate dehydrate (98%), Zinc nitrate hexahydrate (98%) and hexamethylenetetramine (99%) were purchased from Sigma-Aldrich. Whatman filter paper no. 1 (GE-healthcare) was used as the substrate for growing the nanostructures. HIgG and FITC-Ab were purchased from Sigma-Aldrich. Aminopropyltriethoxysilane (APTES) was purchased from Sigma Aldrich. Cardiac myoglobin and anti-myoglobin used in paper-ELISA and control experiments were procured from Abcam. For paper-ELISA we used Human Myoglobin ELISA kit (Abcam, US).

### Hydrothermal growth of ZnO nanorods on Whatman paper

Standard hydrothermal process was used to grow ZnO nanorods on Whatman filter paper no. 1 (WFP) as a substrate. 100 mM solution of zinc acetate (Zn(CH_3_COO)_2_.2H_2_O) was prepared in deionized water and the clean paper (WFP) was soaked in this solution for 60 second followed by annealing at 100 °C for 1 hr to create a seed layer. WFP with the seed layer was then cut into strips of desired dimensions (2 cm * 6 cm) and subjected to further growth process. For the growth of the ZnO nanostructures, the paper strips with the seed layer were transferred to a mini hydrothermal reaction vessel containing equimolar solutions (100 mM, pH-6.5) of hexamethylenetetramine (HMTA) and zinc nitrate Zn(NO_3_)_2_.6H_2_O). The ZnO nanorod synthesis was carried out for 5 hrs at 90 °C. Formation of the Zn(OH)_2_ in a controlled manner is an essential requirement for the growth of the ZnO nanorods. HMTA supplies additional OH^-^ while zinc nitrate is the source of Zn^2+^ ions.

### Characterization of ZnO-NRs/WFP

Structural and surface characterization of the ZnO nanorod modified paper thus generated was performed using a combination of techniques consisting of scanning electron microscopy (SEM), atomic force microscopy (AFM), X- ray diffraction analysis (XRD) and X-ray photon spectroscopy (XPS). The X-ray diffraction data were collected on a RigakuSmartLab X-ray diffractometer using CuKα1 at 40 kV/30 mA, over a range 10° < θ < 60°. Development of nanorods growth on paper was observed and an increase in atomic concentration of Zinc and Oxygen elements was determined by XPS (X-ray photon spectroscopy). For XPS the scan area was kept at 10 μm × 10 μm with analysis in triplicates and the angle of beam relative to paper was 45° for all the measurements.

### Biofunctonalization of ZnO-NRs/WFP

Capture antibodies were immobilized on the ZnO nanorod modified paper using silanization chemistry. For silanization, strips of Whatman filter paper no.1 with ZnO nanorods were dipped into a 1% anhydrous toluene solution of aminopropyltriethoxysilane (APTES) for 5 min. These strips were then heat dried at 100 °C for 15 min. Working solutions (20 μg/mL) were prepared from stock solution of both HIgG (10 μg/mL) and FITC- antiHIgG(1 μg/mL) using 0.1 M PBS (pH-7.4). For antibody immobilization, a freshly prepared antibody solution was drop-casted on the silanized paper and incubated for 1 h in a humid chamber. Finally, to remove loosely bound antibodies, the paper strips were treated with a detergent solution (0.1% aqueous solution of Tween-20) and rinsed with PBS. The non-specific adsorption sites on the antibody immobilized surface were blocked by dipping the paper strips in 1 mg/mL solution of BSA in PBS for one hour at room temperature followed by three rinsing cycles with PBS. For labeling of the immobilized antibody, the paper strips were immersed in a solution of FITC tagged goat antiHIgG (20 μg/mL) and allowed to react for 1hr followed by a thorough wash with PBS. The biofunctionalized ZnO nanorod modified paper strips were stored at 4 °C.

### Fluorescence measurement

To observe protein capture directly on modified paper, the use of fluorescent tagged antibodies was investigated. Confocal microscopy was used to measure and locate the fluorescence of the immobilized antibodies on ZnO-NRs/WFP to avoid the interference of the intrinsic fluorescence of Whatman filter paper. Six types of paper strips were used for the experiment. Two different sets of both modified and unmodified paper strips were made and 5 μL of 10 μg/mL of FITC-Ab was pipetted on the strips. Low concentrations; 50 ng/mL, 100 ng/mL and 200 ng/mL of FITC-Ab were also immobilized on ZnO-NRs/WFP and control paper. To observe the release of the bound antigen (FITC-antiHIgG), a separate experiment was performed using 200 μL of elution buffer (1 mM PB, pH-8.5) which was continuously passed through the paper strips with bound FITC-antiHIgG as antigen and HIgG as capture antibody. The experiment was carried out for 30 min and eluate was collected in 6 different micro-centrifuge tubes at 5 min interval. The experiment was performed in duplicates; Fluorescence spectroscopy was used for quantitative analysis of FITC-antiHIgG in the eluate. The emission and excitation spectra were recorded using a Jasco 2000 spectrofluorometer.

### Paper-based ELISA

To determine the performance of ZnO-NRs/WFP as protein pre-concentrator platform, ELISA was performed on both ZnO nanorods modified and unmodified Whatman filter paper #1. The two types of papers were cut into strips 1 cm × 4 cm and hydrophobic barriers were created using permanent black ink marker (water contact angle 97°). Four identical circular test regions of 5 mm diameter were created on both types of paper strips. Sandwich paper based ELISA (P-ELISA) was performed by immobilizing rabbit polyclonal Anti-myoglobin antibodies on the paper strips as primary antibodies. Biofunctionalization in the circularly marked regions of both the ZnO nanorod modified and unmodified papers was essentially the same as described above followed by blocking with 1 mg/mL BSA solution to avoid non-specific adsorption. The paper strips with the immobilized antibody were used for capture of various concentrations of the marker protein *viz*. Human cardiac Myoglobin. The Abcam ELISA kit with Human Cardiac Myoglobin solutions of 0 ng/mL (control), 50 ng/mL, 100 ng/mL, 250 ng/mL and 500 ng/mL were pipetted on the circular regions having immobilized antibodies on both type of strips. The pipetted volume for each concentration on each circle was 5 μL. Binding of antigen-antibody was allowed for 10 min followed by washing with 10 μL, 1 M PBS three times to remove excess protein. Horse Radish Peroxidase(HRP)-conjugate polyclonal anti-myoglobin was used as secondary labeled antibody. 5 μL of secondary antibody solution was pipetted on each region in both strips and the binding was allowed for 1 min. Then the strips were washed with PBS three times. Now 5 μL of TMB substrate for HRP provided in the kit was placed on each test region and the reaction was allowed to take place for 30 min at room temperature. After 30 min the colour change from colourless to blue was clearly visible. The strips were then scanned using a desktop scanner and analyzed by image J. To show pre-concentration, we prepared 1 mL cardiac myoglobin solution from 250 ng/mL stock and final concentration of the protein solution was 0.5 ng/mL. The entire volume (1 mL) of protein was pipetted drop by drop on each type of strips treated with immobilized antibodies. Similar ELISA experiment was performed on the strips and the colour change was analyzed by image J.

## Additional Information

**How to cite this article**: Tiwari, S. *et al*. Zinc oxide nanorods functionalized paper for protein preconcentration in biodiagnostics. *Sci. Rep.* **7**, 43905; doi: 10.1038/srep43905 (2017).

**Publisher's note:** Springer Nature remains neutral with regard to jurisdictional claims in published maps and institutional affiliations.

## Supplementary Material

Supplementary Information
