# Supplementary material for: Zinc oxide nanorods functionalized paper for protein preconcentration in biodiagnostics
Source: Sci Rep. 2017 Mar 2;7:43905. doi: 10.1038/srep43905 (PMC5333162; doi:10.1038/srep43905)
Supplement: Supplementary Information [file srep43905-s1.pdf]

Zinc oxide nanorods functionalized paper for protein preconcentration in biodiagnostics

Sadhana Tiwari,[1,2] Madhuri Vinchurkar,[1] V. Ramgopal Rao[1] and Gil Garnier[2*]

1. Department of Electrical Engineering, Indian Institute of Technology Bombay, Mumbai 400076, India.
2. BioPRIA, Chemical Engineering department, Monash University Clayton VIC 3800, Australia. * Corresponding author; e-mail: gil.garnier@monash.edu.

S1. EDX spectroscopic study:

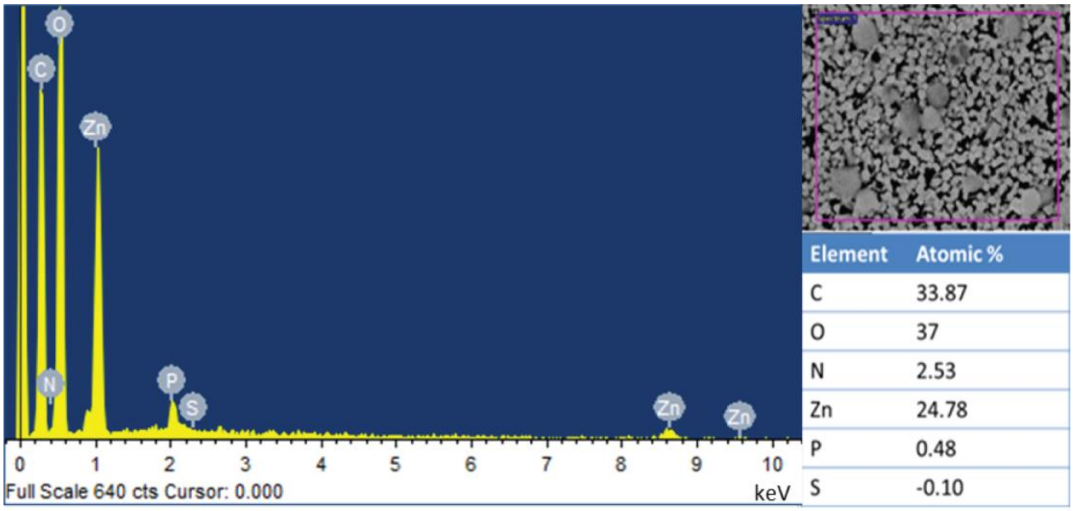

Element	Atomic %
C	33.87
O	37
N	2.53
Zn	24.78
P	0.48
S	-0.10

Figure S1: EDX spectra of ZnO-NRs/WFP showing atomic concentration of different elements.

S2. Nanorods on paper area and density calculation:

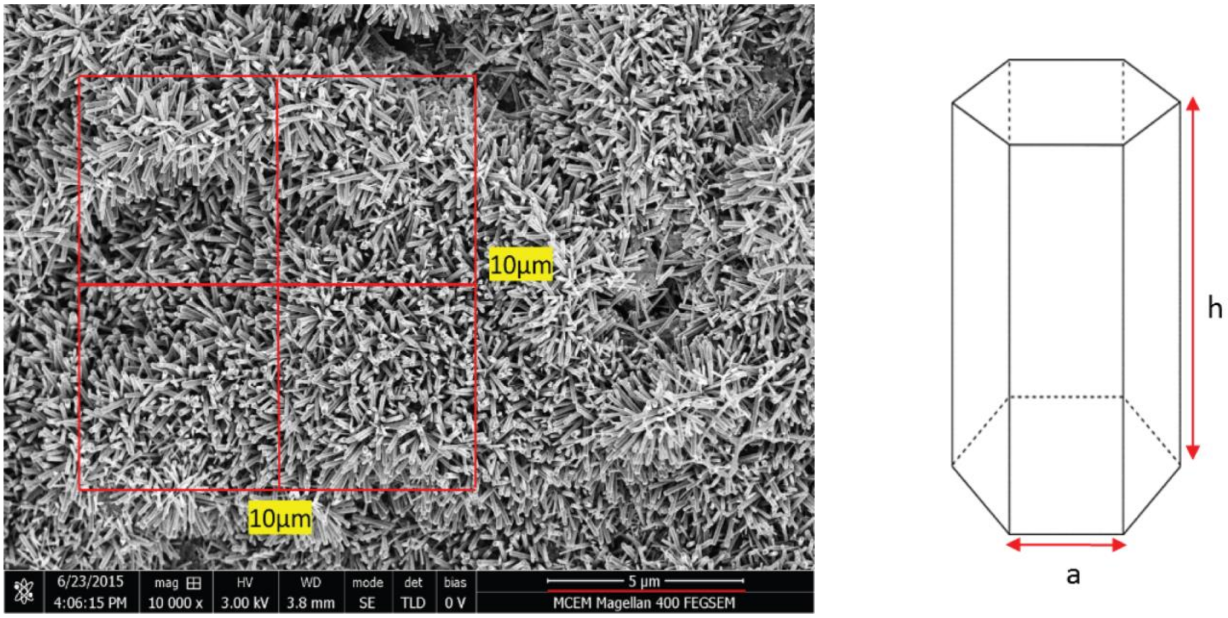

Figure S2: SEM image of ZnO-NRs/WFP and shape of individual rod obtained from SEM and used for calculations.

2.1 Surface area calculation:

Shape of nanorod is like hexagonal prism

Parameter calculated from SEM image:

No. of nanorods in $100 \mu m^2$ is 1000

Height(h): 1µm

Diagonal(D): 200nm

Edge length(a): D/2= 100nm

Area of a hexagonal prism= $3\sqrt{3}a^2 + 6ah$

Hence, area of one nanorod = $6.5 \times 10^{-13}\ m^2 = 0.65 \mu m^2$

Area of 1000 rods will be= $650\ \mu m^2$

2.2 No. of nanorods/m^2 calculation:

Approximate no. of nanorods in $100 um^2$ area is 1000(from SEM image).

No. of rods/m^2 will be:

#rods in $10^{-4}m^2$ is =1000, assuming uniform distribution

In $1m^2 = 1000/10^{-4} = 10^7$ rods/m^2

So, density of rods= 10^7 rods/m^2

Surface coverage of paper by nanorods is about 90% as seen by SEM images.

Therefore, increase in surface area will be= density of rods x area of one rod

$= 6.5 \times 10^{-13} \times 10^7$

$= 6.5\text{x } 10^{-6}\ m^2$ or $6.5\text{x } 10^6 \mu m^2$

$= 7\text{x}10^6\ \mu m^2$

S3. Relation with increase in fluorescence

**Sample**	**Average Intensity**
**WFP**	6440184
**ZnO-NRs/WFP**	22101495

Percent increase in fluorescence intensity=

$$\frac{FinalIntensity - InitialIntensity}{InitialIntensity} x100$$

Which gives,

= 22101495-6440184/6440184*100

> 200% increase in fluorescence.
